# NUSAP1 Binds ILF2 to Modulate R-Loop Accumulation and DNA Damage in Prostate Cancer

**DOI:** 10.3390/ijms24076258

**Published:** 2023-03-26

**Authors:** Chun-Lung Chiu, Caiyun G. Li, Erik Verschueren, Ru M. Wen, Dalin Zhang, Catherine A. Gordon, Hongjuan Zhao, Amato J. Giaccia, James D. Brooks

**Affiliations:** 1 Department of Urology, Stanford University School of Medicine, Stanford, CA 94305, USA; 2 Department of Radiation Oncology, Stanford University School of Medicine, Stanford, CA 94305, USA; 3 ULUA Besloten Vennootschap, Arendstraat 29, 2018 Antwerpen, Belgium; 4 Medical Research Council/Cancer Research United Kingdom Oxford Institute for Radiation Oncology and Gray Laboratory, University of Oxford, Oxford OX3 7DQ, UK; 5 Stanford Cancer Research Institute, Stanford University School of Medicine, Stanford, CA 94305, USA

**Keywords:** NUSAP1, ILF2, DHX9, R-loop, DNA damage, prostate cancer

## Abstract

Increased expression of NUSAP1 has been identified as a robust prognostic biomarker in prostate cancer and other malignancies. We have previously shown that NUSAP1 is positively regulated by E2F1 and promotes cancer invasion and metastasis. To further understand the biological function of NUSAP1, we used affinity purification and mass spectrometry proteomic analysis to identify NUSAP1 interactors. We identified 85 unique proteins in the NUSAP1 interactome, including ILF2, DHX9, and other RNA-binding proteins. Using proteomic approaches, we uncovered a function for NUSAP1 in maintaining R-loops and in DNA damage response through its interaction with ILF2. Co-immunoprecipitation and colocalization using confocal microscopy verified the interactions of NUSAP1 with ILF2 and DHX9, and RNA/DNA hybrids. We showed that the microtubule and charged helical domains of NUSAP1 were necessary for the protein-protein interactions. Depletion of ILF2 alone further increased camptothecin-induced R-loop accumulation and DNA damage, and NUSAP1 depletion abolished this effect. In human prostate adenocarcinoma, *NUSAP1* and *ILF2* mRNA expression levels are positively correlated, elevated, and associated with poor clinical outcomes. Our study identifies a novel role for NUSAP1 in regulating R-loop formation and accumulation in response to DNA damage through its interactions with ILF2 and hence provides a potential therapeutic target.

## 1. Introduction

Approximately 268,000 men will be diagnosed with prostate cancer in the U.S. in 2022 and 34,500 will die from their disease [1]. The large discrepancy between the incident and mortality rates points to the clinical challenge of distinguishing men who need treatment from those who can be safely managed with active surveillance. Increased expression of nucleolar and spindle-associated protein 1 (NUSAP1) has been identified as a robust clinical biomarker of aggressive prostate cancer and is part of two commercial prognostic gene expression panels used to analyze prostate tissue samples [2,3,4,5]. Over the past decade, increased expression of NUSAP1 has been shown to be associated with poor outcomes in many solid tumors, including those of the skin [6,7], breast [8,9,10,11], prostate [2,3,12], cervix [13,14], brain [15,16,17], liver [18,19,20], pancreas [21,22], stomach [23,24], ovary [25,26] and lung [27,28].

NUSAP1 associates with the mitotic spindle and contains DNA and microtubule-binding domains [29,30]. It binds to the mitotic spindle and participates in chromosome segregation by regulating the kinetochore during mitosis [29,30]. NUSAP1 is expressed in the nucleolus during interphase and is expressed throughout the cell cycle, with expression levels peaking in G2/M phase [30]. NUSAP1 expression is increased by E2F1, and allelic loss of the retinoblastoma gene, a common finding in prostate cancer, can also increase NUSAP1 expression [2,31]. Given its role in mitosis, a logical assumption would be that overexpression of NUSAP1 is merely a surrogate for more highly mitotic cancers, and high proliferation rates are associated with poor outcomes in most malignancies. However, forced overexpression of NUSAP1 does not affect prostate cancer cell growth in vitro or in vivo [32]. Elevated NUSAP1 expression increases cell motility and invasion in prostate cancer and astrocytoma cells and metastasis in xenograft models [17,32].

The mechanisms by which NUSAP1 contributes to cancer progression, metastases, and poor outcomes are poorly understood. NUSAP1 has been linked to chromosome instability and DNA damage response (DDR) pathways through upregulating BRCA1 expression and recruitment to DNA damage foci in breast cancer and through activation of Ataxia telangiectasia and Rad3-related protein (ATR) in glioblastoma [33,34]. In addition, NUSAP1 has been shown to promote metastasis by activating WNT signaling pathways in cervical cancer, triple-negative breast cancer, and nasopharyngeal carcinoma, and Hedgehog signaling in astrocytoma [8,17,35,36]. Increased NUSAP1 expression has also been shown to enhance PI3K/AKT signaling [37]. While these downstream pathways could contribute to cancer aggressiveness, we speculated that NUSAP1 protein has additional means of driving progression through its interactions with protein-binding partners. To identify candidate interacting proteins, we performed affinity purification–mass spectrometry (AP–MS) and validation of candidate binding partners to identify a novel role for NUSAP1 in the regulation of R-loops and DNA damage in prostate cancer.

## 2. Results

### 2.1. AP–MS Identifies NUSAP1 Interactions with ILF2 and DHX9

**Figure 1:**
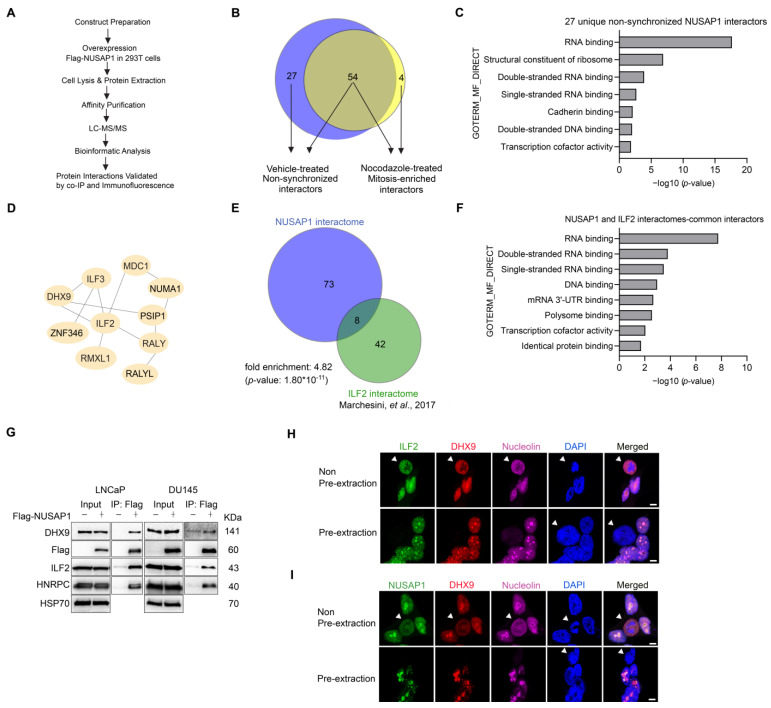
Identification of NUSAP1 interactors, ILF2, and DHX9 by AP–MS. (**A**) Flowchart of AP–MS and verification of protein-protein interactions by co-IP and immunofluorescence. (**B**) Venn diagram of 85 Flag-NUSAP1 interacting proteins identified by AP–MS. Nocodazole induced mitotic arrest of 58 mitosis-enriched proteins while non-synchronized cells (vehicle) identified 81 proteins of which 27 interactors were not mitosis associated. (**C**) Gene ontology (GO) analysis of the molecular functions (MF) of the 27 non-synchronized NUSAP1 interactors by DAVID are enriched for RNA and DNA binding. (**D**) Ingenuity Pathway Analysis (IPA) reveals NUSAP1 interaction with an ILF2/DHX9 protein network. (**E**) Significant overlap 8 interactors between the NUSAP1 and ILF2 interactomes [40] including ILF2 and DHX9. (**F**) Gene ontology (GO) analysis of the molecular functions (MF) of the 8 common interactors between NUSAP1 and ILF2 interactomes by DAVID are enriched for RNA and DNA binding. (**G**) Immunoprecipitation and western blots validate DHX9, ILF2, and HNRPC interactions with Flag-NUSAP1. (**H**) Confocal microscopy shows ILF2 and DHX9 under conventional and pre-extraction conditions. For pre-extraction, cells were permeabilized by 0.5% Triton X-100 before PFA fixation. Under pre-extraction, ILF2 and DHX9 are visualized mainly in the nucleolus, shown by staining with nucleolin. (**I**) NUSAP1 and DHX9 co-localize with nucleolin in LNCaP cells. Arrowheads indicate mitotic cells. Scale bar = 10 µm.

Based on our previous demonstration that NUSAP1 overexpression increases prostate cancer cell invasion, migration, and metastasis [2,31,32], we focused on identifying its protein-binding partners using the AP-MS analysis approach outlined in Figure 1A. We selected 293T cells because of the high transfection efficiency and their tolerance to NUSAP1 overexpression. Notably, 293T cells were transiently transfected with an empty vector or Flag-tagged NUSAP1 (Flag-NUSAP1) constructs and then treated with nocodazole or DMSO (the vehicle) for 24 h prior to pulldown with an anti-Flag antibody in the presence of micrococcal nuclease. Affinity pulldown experiments were performed in non-synchronized cells and cells treated with nocodazole to induce G2/M cell cycle arrest [38] to enrich NUSAP1 mitotic interacting partners. Putative interacting proteins were identified using SAINT (Significance Analysis of INTeractome) [39] to select the high-confident NUSAP1-interacting proteins (SAINT Score > 0.9 and BFDR < 0.05) based on triplicate pulldown experiments compared against the respective vehicle controls. In the non-synchronized cells, 81 putative interactors (Table S1) were identified while 58 putative interactors (Table S2) were found in the nocodazole-treated cells (Figure 1B; Tables S1 and S2).

Since the effects of NUSAP1 on prostate cancer invasion, motility, and metastases reflect non-mitotic cell functions [32], we focused on the 27 NUSAP1-interacting proteins (Table S3) uniquely identified in the non-synchronized cells. Using the DAVID Gene Ontology (GO) analysis tool, we found that RNA binding was among the most highly enriched molecular functions (Figure 1C). Furthermore, Ingenuity Pathway Analysis (IPA) identified NUSAP1 as part of the ILF2 network, which included ILF2, ILF3, DHX9, RMXL1, ZNF346, MDC1, NUMA1, PSIP1, RALY and RALYL (Figure 1D, Table S3). To understand the potential relationship between NUSAP1 and ILF2, we compared our NUSAP1 interactome to a previously published ILF2 interactome [40] and found an overlap of eight interacting proteins, including DHX9, ILF2, ILF3, HNRPC, RALY, FUS, RBMX, and YBOX3 (Figure 1E, Table S4). DAVID analysis of those eight interacting proteins also demonstrated enriched molecular functions of RNA and DNA binding (Figure 1F).

ILF2 and DHX9 have separately been found to associate with R-loops [41,42,43], which are DNA/RNA hybrids implicated in transcription, cellular stress, and DNA repair, particularly homologous recombination [44,45]. Since genomic rearrangements have been observed in prostate cancers [46,47,48], we sought to investigate further the interactions of these R-loop-associated proteins and NUSAP1 in prostate cancer cells. To confirm the interaction of NUSAP1 and ILF2, DHX9, and HNRPC, we transiently transfected LNCaP and DU145 prostate cancer cell lines with empty vector or Flag-NUSAP1. Using an anti-Flag antibody, we showed that Flag-NUSAP1 co-immunoprecipitated with ILF2, DHX9, and HNRPC (Figure 1G). Immunofluorescence staining of non-transfected LNCaP cells demonstrated localization of DHX9, ILF2, and native NUSAP1 in the nucleoplasmic region and the nucleoli (marked by nucleolin staining) (Figure 1H,I). We further defined the nucleolar localization of these proteins using the Triton-X pre-extraction staining method followed by confocal microscopy (Figure 1H,I) [30]. These results indicate that NUSAP1 physically binds DHX9 and ILF2 in the nucleolus.

### 2.2. NUSAP1 MT and ChHD Domains Are Important for ILF2 and DHX9 Interactions

**Figure 2:**
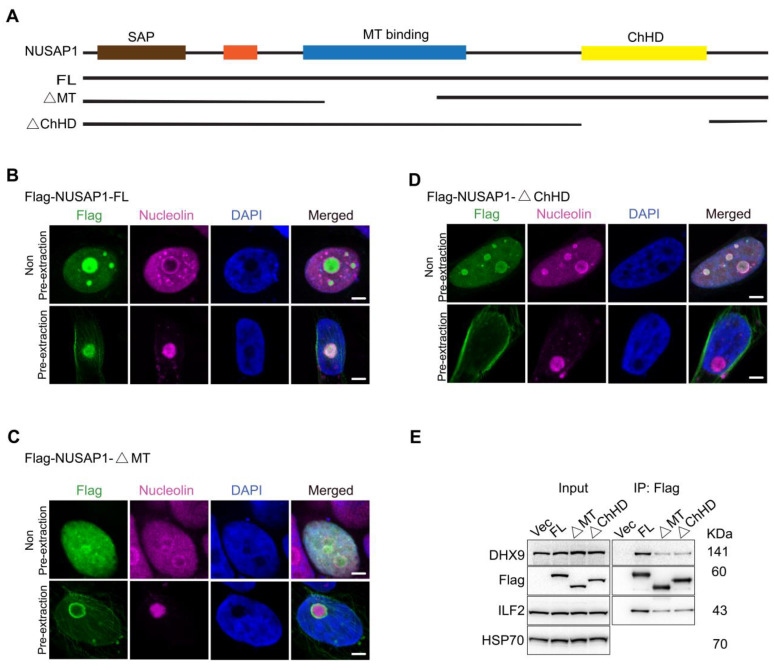
NUSAP1 interactions with DHX9 and ILF2 require MT and ChHD domains. (**A**) Summary of the full-length Flag-NUSAP1 (FL), MT domain (fragment 243–367) deletion Flag-NUSAP1 (ΔMT) and ChHD deletion Flag-tagged NUSAP1 (ΔChHD) fragment plasmids. (**B**–**D**) Representative confocal microscopy images of localization of Flag-NUSAP1-FL (**B**), -ΔMT (**C**), and -ΔChHD (**D**). Scale bar = 10 µm. (**E**) IP-WB shows decreased binding of Flag-NUSAP1-ΔMT and ΔChHD mutants to DHX9 and ILF2 compared to Flag-NUSAP1-FL in LNCaP cells.

The NUSAP1 protein harbors a DNA-binding SAP domain, as well as a microtubule (MT) binding domain and the charged helical domain (ChHD) [29,30]. To understand the binding properties of NUSAP1 with ILF2 and DHX9, we examined whether NUSAP1 interacts with ILF2 and DHX9 through its MT or ChHD domains by transfecting LNCaP cells with Flag-NUSAP1 full-length (FL), Flag-NUSAP1 with a partial deletion of the MT-domain (ΔMT), or Flag-NUSAP1 with a deletion of the ChHD domain (ΔChHD) (Figure 2A). We first used confocal microscopy in LNCaP cells expressing Flag-NUSAP1-FL, -ΔMT, and -ΔChHD to validate cellular localizations of the wild-type and mutant NUSAP1. As expected, with pre-extraction, Flag-NUSAP1-FL was localized in the nucleoli, as judged by the colocalization with nucleolin staining (Figure 2B). However, Flag-NUSAP1-ΔMT was found at the periphery of the nucleoli and was probably not overlapping (Figure 2C) with ILF2 and DHX9 (Figure 1H,I). In comparison, Flag-NUSAP1-ΔChHD lost its nucleolar localization entirely and was found at the periphery of the nucleus (Figure 2D) under pre-extraction conditions, implying that the ChHD domain is critical for NUSAP1 nuclear localization and its colocalization with ILF2 and DHX9. We next conducted immunoprecipitation using the anti-Flag antibody from whole-cell extracts and demonstrated diminished levels of ILF2 and DHX9 being pulled down when either the MT or ChHD domains were deleted compared to full-length NUSAP1 (Figure 2E). These data showed that both MT and ChHD domains are required not only for NUSAP1 cellular localization, but they are also essential for its interactions with ILF2 and DHX9.

### 2.3. NUSAP1, ILF2 and DHX9 Associate with R-Loops

**Figure 3:**
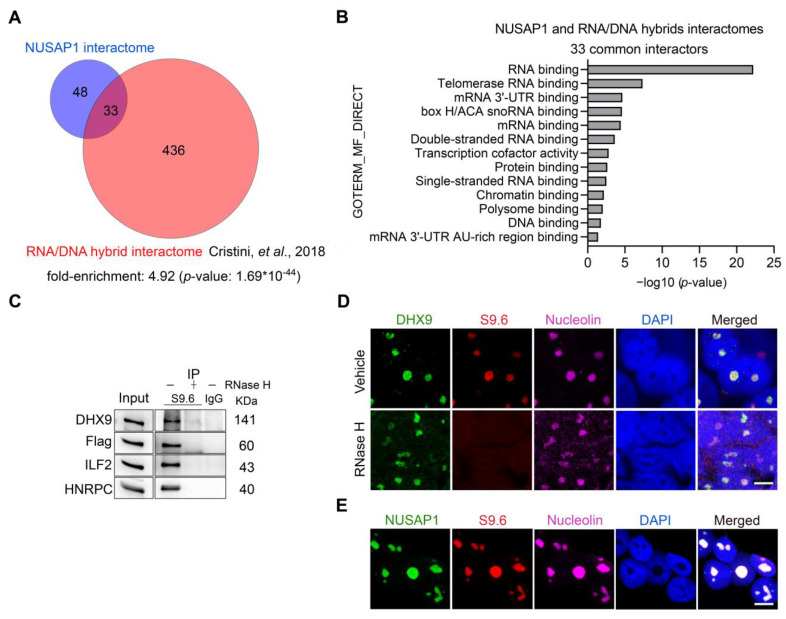
NUSAP1, ILF2, and DHX9 interact with R-loops. (**A**) Venn diagram of 33 proteins found in both NUSAP1 and RNA/DNA hybrid interactomes [41], including ILF2 and DHX9. (**B**) Gene ontology (GO) analysis of the 33 common NUSAP1 and R-loop-interacting proteins shows enrichment for RNA and telomerase RNA binding. (**C**) Immunoprecipitation with S9.6 antibody that binds specifically to RNA/DNA hybrids confirms interactions with DHX9, Flag-NUSAP1, ILF2, and HNRPC. Binding was abolished by pretreatment with RNase H, confirming specificity of their interactions with R-loops. (**D**,**E**) Confocal microscopy images of pre-extracted LNCaP cells for native NUSAP1, DHX9, nucleolin, and R-loops (S9.6) demonstrate colocalization. Scale bar = 10 µm.

Given previous evidence demonstrating that ILF2 and DHX9 are associated with R-loops [41,43], we tested whether NUSAP1 is associated with these RNA/DNA hybrids. We compared our NUSAP1 interactome with an R-loop interactome generated by immunoprecipitation using the S9.6 antibody that specifically binds RNA/DNA hybrids [41] and found a significant overlap of 33 common proteins (Figure 3A, Table S5), including ILF2, DHX9, and HNRPC. Pathway analysis showed significant enrichment for RNA-binding proteins and the telomere (Figure 3B and Figure S1A,B), where RNA/DNA hybrids are known to be present [49]. To determine whether NUSAP1, ILF2, DHX9, and HNRPC are associated with R-loops in prostate cancer cells, we performed immunoprecipitation using the S9.6 antibody in LNCaP cells. Immunoblotting confirmed an association of NUSAP1, ILF2, DHX9, and HNRPC with R-loops (Figure 3C). Treatment of cell extracts with RNase H, which specifically digests RNA/DNA hybrids [41], eliminated pulldown and detection of these proteins, demonstrating that they associate with R-loops specifically (Figure 3C). Immunofluorescence imaging with confocal microscopy demonstrated colocalization of native NUSAP1, DHX9, nucleolin, and S9.6 antibody staining, while RNase H treatment diminished the nuclear fluorescence intensity of S9.6, confirming that these proteins are associated with R-loop structures (Figure 3D,E).

### 2.4. NUSAP1 Depletion Reduces CPT-Induced R-Loop Accumulation and DNA Damage but Does Not Compensate for DHX9 Depletion

**Figure 4:**
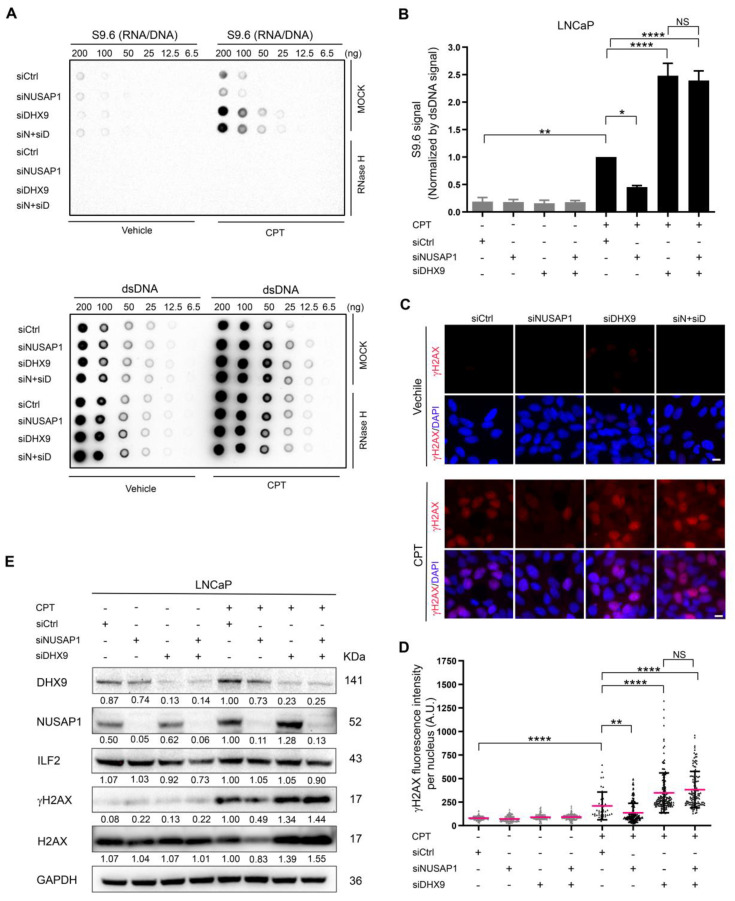
NUSAP1 depletion reduces CPT-induced R-loop accumulation and DNA damage and DHX9 depletion increases both. (**A**) Dot-blot analysis of R-loop levels in LNCaP cells without and with 10 μM Camptothecin (CPT) treatment for 60 min after 48 h post-transfection of siNUSAP1, siDHX9, or both. Serial dilutions of RNA/DNA hybrids (R-loops) and dsDNA extracts were spotted on the membrane for each condition. R-loop formation was increased with CPT treatment. siNUSAP1 alone reduced R-loop levels, while siDHX9 significantly increased R-loops. siDHX9 and siNUSAP1 together did not reduce the accumulation of R-loops compared to siDHX9 alone. Bottom panels anti-DS DNA antibody serves as loading control. (**B**) S9.6 signal from the dot-blots in the 100 ng nucleic acid samples normalized by dsDNA signal quantified using Image J (*n* = 3). (**C**) Immunofluorescent staining using anti-*γ*H2AX (red) to assess DNA damage without and with CPT treatment in LNCaP cells. siNUSAP1 decreases *γ*H2AX staining while siDHX9 increases staining significantly, which is not affected by siNUSAP1. DAPI (blue). Scale bar = 10 µm. (**D**) *γ*H2AX fluorescence intensities quantified by Image J (*n* = 3). (**E**) Representative Western blot analysis of NUSAP1, ILF2, DHX9, H2AX, and *γ*H2AX protein levels with CPT and the respective siRNA treatments (*n* = 3). siCtrl, siControl; siN + siD, siNUSAP1 + siDHX9. *, *p* < 0.05; **, *p* < 0.01; ****, *p* < 0.0001; NS, *p* > 0.05.

Camptothecin (CPT), a topoisomerase I inhibitor, promotes R-loop accumulation and causes unresolved DNA supercoiling, thereby promoting DNA damage [41,50,51]. Knockdown of DHX9 expression has been reported to increase R-loops and DNA damage [41]. To assess whether the interactions between NUSAP1 and DHX9 affect R-loop levels and DNA damage, we used small interfering RNA (siRNA) to deplete NUSAP1 and DHX9, either alone or in combination, in LNCaP cells with or without CPT treatment. R-loop levels were assessed by probing dot-blots of nucleic acid extracts with the S9.6 antibody, and the specificity was confirmed by pretreatment with RNase H to digest R-loops (Figure 4A). Without CPT treatment, R-loop levels are minimal and unaffected by depletion of NUSAP1, DHX9, or both (Figure 4A,B). After treatment with CPT, R-loop levels increased significantly in LNCaP vector controls, and depletion of NUSAP1 decreased R-loop accumulation (Figure 4A,B). As expected, the depletion of DHX9 significantly increased R-loop levels [41]; however, the depletion of NUSAP1 in addition to DHX9 did not compensate for DHX9 depletion (Figure 4A,B).

Depletion of DHX9 has also been associated with a significant increase in CPT-induced DNA damage due in part to the increase in R-loops [41,52]. Without CPT treatment, DNA damage levels, assessed by nuclear staining for *γ*H2AX, remained low, regardless of knockdown of NUSAP1, DHX9, or both (Figure 4C,D). In the presence of CPT, *γ*H2AX staining increased significantly, and the knockdown of NUSAP1 significantly decreased *γ*H2AX staining, paralleling its effect on R-loop levels (Figure 4C,D). Depletion of DHX9 dramatically increased *γ*H2AX staining upon CPT treatment and was not affected by co-depletion of NUSAP1. *γ*H2AX protein levels under these knockdown conditions were analyzed by Western blot analysis. Following CPT treatment, NUSAP1 depletion alone decreased *γ*H2AX protein levels by half, while DHX9 depletion increased *γ*H2AX levels and was not affected by NUSAP1 co-depletion (Figure 4E). Therefore, upon CPT-induced DNA damage, NUSAP1 appears to promote R-loop and DNA damage accumulations, while DHX9 is required for resolving DNA damage and R-loop formation and might compensate for R-loop formation and DNA damage induced by NUSAP1.

### 2.5. ILF2 Depletion Increases R-Loop Accumulation and DNA Damage That Is Abolished by Depletion of NUSAP1

**Figure 5:**
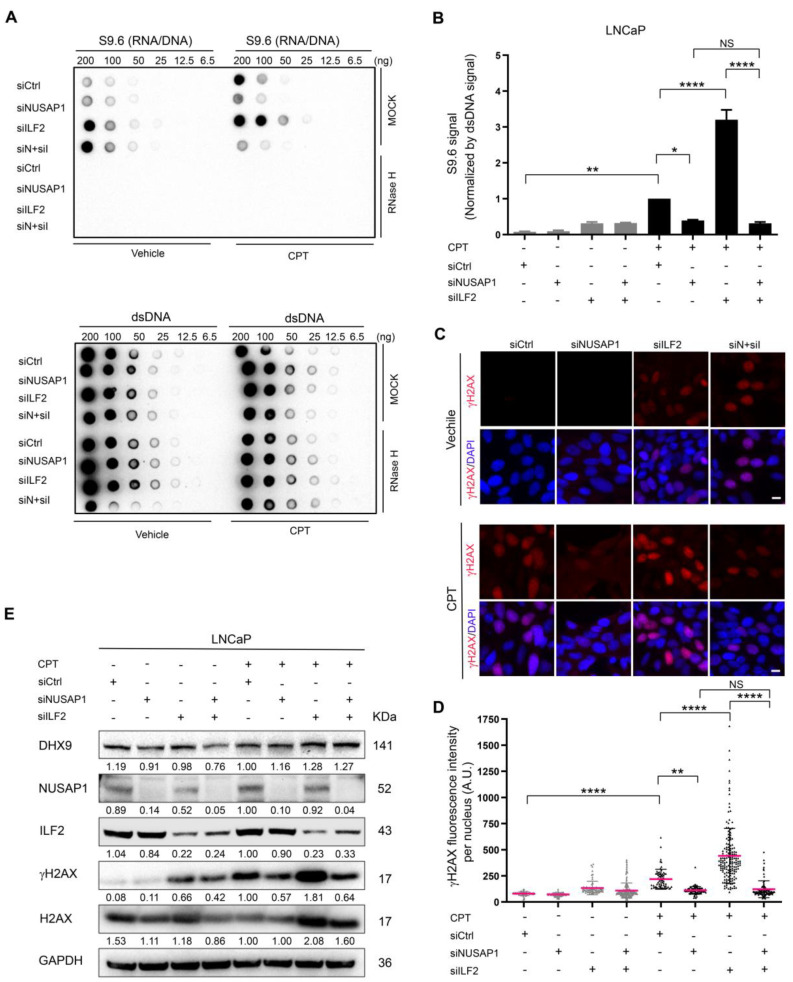
ILF2 depletion promotes R-loop accumulation and DNA damage which are prevented by NUSAP1 depletion. (**A**) Dot-blot analysis of R-loop levels in LNCaP cells without and with 10 μM Camptothecin (CPT) treatment for 60 min after 48 h post-transfection of siNUSAP1, siILF2 or both. siNUSAP1 alone reduced R-loop levels and siILF2 significantly increased R-loops. siNUSAP1 and siILF2 together completely corrected R-loop levels compared to siILF2 alone. Bottom panels anti-DS DNA antibody serves as loading control. (**B**) S9.6 signal from the dot-blots in the 100 ng nucleic acid samples normalized by dsDNA signal quantified using Image J (*n* = 3). (**C**) Immunofluorescent staining using anti-*γ*H2AX (red) to assess DNA damage without and with CPT treatment in LNCaP cells. siNUSAP1 decreases *γ*H2AX staining while siILF2 increases staining significantly. siNUSAP1 reduces *γ*H2AX levels to baseline in the presence of siILF2. DAPI (blue). Scale bar = 10 µm (**D**) *γ*H2AX fluorescence intensities quantified by Image J (*n* = 3). (**E**) Representative Western blot analysis of NUSAP1, ILF2, DHX9, H2AX, and γH2AX protein levels (*n* = 3). siCtrl, siControl; siN + siI, siNUSAP1 + siILF2. *, *p* < 0.05; **, *p* < 0.01; ****, *p* < 0.0001; NS, *p* > 0.05.

ILF2 promotes DDR by modulating YB-1 nuclear translocation to regulate mRNA splicing of DDR proteins [40]. ILF2 has been reported to associate with R-loops, although its functional role in R-loop biology, particularly related to R-loop-mediated DNA damage, is unknown [43]. Depletion of ILF2 alone or in combination with NUSAP1 depletion slightly increased R-loop formation at baseline as assessed by the dot-blot analysis (Figure 5A,B). Following CPT treatment, depletion of ILF2 significantly increased R-loop formation, while depletion of NUSAP1 decreased R-loops (Figure 5A,B). Strikingly, the depletion of NUSAP1 and ILF2 together eliminated the increase in R-loops seen with ILF2 depletion alone, restoring R-loop levels to near baseline (Figure 5A,B). Depletion of ILF2 showed similar effects on DNA damage as measured by nuclear *γ*H2AX staining. In the absence of CPT, the depletion of ILF2 slightly increased *γ*H2AX staining levels. Following CPT treatment, NUSAP1 depletion alone decreased *γ*H2AX protein levels, while depletion of ILF2 alone increased *γ*H2AX dramatically (Figure 5C,D). The effect of IL2 depletion was eliminated with the co-depletion of NUSAP1 (Figure 5C,D). *γ*H2AX levels were confirmed by western blot, where deletion of ILF2 levels alone significantly increased *γ*H2AX levels, while deletion of ILF2 and NUSAP1 simultaneously decreased *γ*H2AX levels similar to those seen with depletion of NUSAP1 alone in cells treated with CPT (Figure 5E). Together, these data demonstrate that ILF2 appears to prevent R-loop and DNA damage accumulation and this effect depends on its binding partner NUSAP1.

### 2.6. High NUSAP1 Expression Correlated with DNA Repair Pathways and Poor Clinical Outcome in Prostate Adenocarcinoma

**Figure 6:**
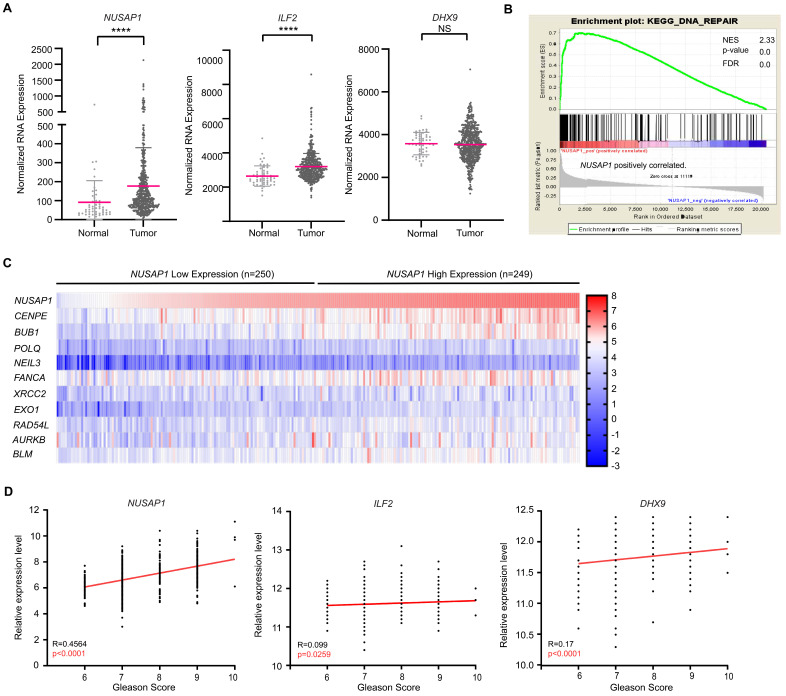

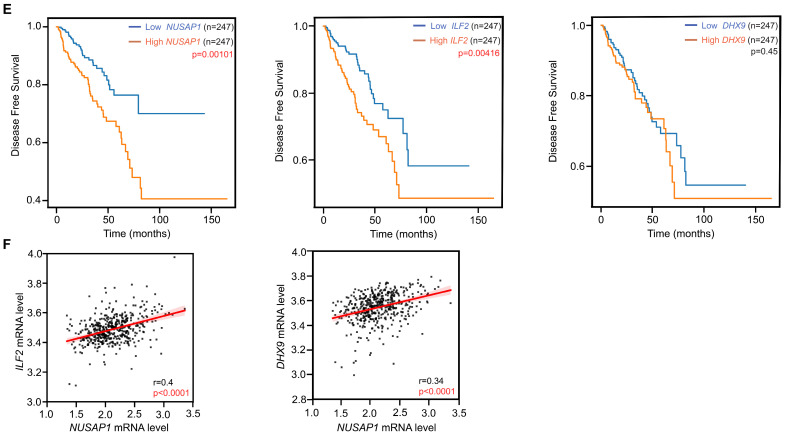
High *NUSAP1* and *ILF2* expression correlated with DNA repair pathways and adverse clinical features in prostate adenocarcinoma. (**A**) *NUSAP1* mRNA levels were significantly higher in prostate adenocarcinoma compared to non-cancerous prostate tissues in the TCGA PRAD dataset. (**B**) GSEA demonstrated *NUSAP1* mRNA level was positively correlated with the DNA repair pathway. (**C**) Heatmap of genes showing high correlation with *NUSAP1* transcript levels. (**D**) *NUSAP1* mRNA levels are associated with tumor grade (Gleason scores) while *ILF2* and *DHX9* mRNA levels were less correlated with tumor grade. (**E**) Kaplan–Meier analysis shows that increased *NUSAP1* and *ILF2* transcript levels, divided at the median, are associated with disease-specific survival; *DHX9* expression levels are not associated with worse outcomes (*p*-values by Log-rank test). (**F**) Pearson correlation coefficient analysis revealed that *NUSAP1* mRNA level was significantly correlated with *DHX9* and *ILF2* mRNA levels. ****, *p* < 0.0001; NS, *p* > 0.05.

Increased NUSAP1 expression has been associated with adverse clinical outcomes in many cancers. In prostate cancer, NUSAP1 increases invasion, cell migration, and metastasis [2,32]. In The Cancer Genome Atlas (TCGA) Prostate Adenocarcinoma (PRAD) dataset [48], *NUSAP1* mRNA levels are upregulated in prostate adenocarcinoma tissues compared with non-cancerous prostate tissues (Figure 6A). Gene Set Enrichment Analysis (GSEA) showed that high expression of *NUSAP1* was positively associated with DNA repair-related signaling pathway (Figure 6B), and high expression of *NUSAP1* was correlated with DDR-associated genes, such as *POLQ*, *NEIL3*, *FANCA*, *XRCC2*, *EXO1*, *RAD54L*, and *BLM* (Figure 6C). In addition, increased *NUSAP1* and *DHX9* mRNA levels were significantly correlated with higher tumor Gleason scores, while increased *ILF2* were less correlated with tumor Gleason scores (Figure 6D). Kaplan–Meier analysis demonstrated that cancers with high *NUSAP1* and *ILF2* mRNA levels had a significantly worse disease-free survival rate (Figure 6E). Interestingly, *DHX9* expression levels were not associated with recurrence after surgery (Figure 6E). Furthermore, *NUSAP1* mRNA levels correlated significantly with *ILF2* and *DHX9* mRNA levels (*p* < 0.0001, Pearson correlation coefficient) (Figure 6F). Therefore, human localized prostate cancer samples show co-expression of *NUSAP1* with its binding partners, *ILF2* and *DHX9*. Increased *NUSAP1* and *ILF2* expression were both associated with adverse outcomes, while *DHX9* expression was not, mirroring in vitro data showing strong cooperation between *NUSAP1* and *ILF2* with R-loop and DNA damage pathways.

## 3. Discussion

To better understand the mechanisms underlying the association of NUSAP1 overexpression and cancer aggressiveness, we used AP–MS to identify novel interactions between NUSAP1 and several proteins with described functions in R-loop biology and DDR, including DHX9 [41,53], ILF2 [40,43,54], HNRPC [55,56], FUS [57,58,59], and RBMX [60,61]. We experimentally validated that NUSAP1 co-localizes with and binds to DHX9 and ILF2 and demonstrated direct binding of NUSAP1 to RNA/DNA hybrid R-loops. Furthermore, we show that depletion of NUSAP1 by itself decreases R-loops and DNA damage in cells treated with CPT. R-loops have been implicated in promoting DNA damage and genomic instability [42,44,45,62], suggesting that elevated levels of NUSAP1 could drive cancer aggressiveness by increasing genomic instability. In prostate cancer and other malignancies, an increased burden of DNA structural alterations has been associated with adverse outcomes [46]. A recent pan-cancer analysis confirms that increased levels of DNA structural alterations are associated with advanced cancer stages in many cancers, including those where increased NUSAP1 expression levels are associated with adverse outcomes [63]. Whether NUSAP1 overexpression directly affects DNA structural alterations, particularly through R-loop mediated mechanisms, require a deeper understanding of the linkage between R-loops and DNA damage [42].

The effects of NUSAP1, R-loop abundance, and DNA damage appear to be mediated, at least in part, through its interaction with ILF2. ILF2 had been previously identified by MS to be associated with RNA/DNA hybrids [43] and has been shown to promote DNA repair through post-transcriptionally modulating DNA damage-induced splicing in multiple myeloma [40]. We are the first to demonstrate that depletion of ILF2 increases R-loop abundance and DNA damage in the absence and presence of CPT. More importantly, the interaction of ILF2 with NUSAP1 appears to be critical in mediating these effects since depletion of NUSAP1 corrects R-loop and *γ*H2AX accumulation induced by depletion of ILF2. While ILF2 predominantly binds to RNA, NUSAP1 can bind to DNA, both single-stranded and double-stranded through its SAP domain [29]. As cells transition through mitosis, NUSAP1 stabilizes and cross-links microtubules and may tether microtubules to chromosomes [64,65]. This unique function of simultaneously binding to both proteins and nucleic acid structures could also apply to its relationship with ILF2 or DHX9 and R-loops. In addition to our demonstration of colocalization and binding of NUSAP1 and ILF2 by western blotting, several pathways have been implicated in R-loop regulation, and our data indicate that NUSAP1, ILF2, and possibly other NUSAP1 interacting proteins are potential pathways that regulate R-loop abundance [42].

The role of NUSAP1 in DDR remains unclear, although it has been physically linked to the two key kinases, ATM and ATR, in the DDR signaling cascade [34,66]. NUSAP1 stabilizes ATR protein by promoting the sumoylation of ATR, while it is a phosphorylation substrate of ATM [66]. It would be critical to understand whether NUSAP1 phosphorylation by means of ATM and its sumoylation activity are associated with its functions in altering R-loops and its interactions with ILF2 or DHX9. Both NUSAP1 MT and ChHD domains appear to be important for maintaining NUSAP1 nuclear distribution and mediating the interaction between NUSAP1 and ILF2/DHX9. Future work will focus on identifying the regions of ILF2 and DHX9 necessary for their interactions with NUSAP1 and for understanding if these motifs are critical in determining NUSAP1 nuclear distribution, especially upon DNA damage.

Analysis of NUSAP1, ILF2, and DHX9 in the TCGA dataset parallels our findings, substantiating the important role of the NUSAP1 and ILF2 interaction in prostate cancer aggressiveness. Overexpression of *NUSAP1* and *ILF2* are both upregulated in cancer compared to non-cancerous prostate tissues and both correlate with an increased risk of recurrence after surgery. Likewise, increased expression of *NUSAP1* and *ILF2* are correlated with higher Gleason scores of cancer, the most important clinical predictor of recurrence [67]. *DHX9* expression, however, does not correlate with recurrence, despite correlating with the Gleason score. *NUSAP1* expression levels show a positive correlation with the expression of both *DHX9* and, to a greater degree, with *ILF2*, demonstrating that they are coregulated, as would be expected for interacting proteins. The mechanisms through which NUSAP1 and ILF2 interact to affect prognosis are not clear. In multiple myeloma, increased ILF2 is associated with therapeutic resistance mediated through DNA repair pathways [40], and it is interesting to note that increased expression of NUSAP1 is associated with DNA repair pathways in the PRAD data in our study and others [3]. Whether the increased expression of ILF2 is a reaction to or a partner in R-loop formation and DNA damage induced by increased NUSAP1 expression is an interesting question which we are investigating further.

We propose that increased expression of NUSAP1 and ILF2 drive progression, at least in part through their effects on R-loops and DNA damage; however, it is possible that this is not the major pathway through which these proteins increase cancer aggressiveness. Indeed, many of the NUSAP1 interacting proteins have been implicated in RNA binding, splicing, and transport and it is possible these pathways are the primary drivers of cancer aggressiveness [68]. Regardless, our study demonstrates that NUSAP1 interacts with ILF2 and DHX9 and is involved in R-loop biology. Since high expression of NUSAP1 is positively associated with R-loop accumulation and DNA damage, one therapeutic strategy could entail blocking DNA damage repair with poly (ADP-ribose) polymerase (PARP) inhibitors in cancers with high-level expression of NUSAP1. In other words, the increased DNA damage arising from R-loops in cancers with high NUSAP1 expression could represent a therapeutic vulnerability that could be exploited by blocking DNA repair using PARP inhibition.

## 4. Materials and Methods

### 4.1. Cell Culture

293. T cells (ATCC) and DU145 cells were maintained in Dulbecco’s Modified Eagle’s Medium (DMEM, GIBCO, Waltham, MA, USA), and LNCaP cells were maintained in Roswell Park Memorial Institute 1640 Medium (RPMI-1640, GIBCO), supplemented with 10% fetal bovine serum (FBS, GIBCO), 1% penicillin/streptomycin (GIBCO), and incubated at 37 °C with 5% CO_2_.

### 4.2. siRNA

ON-TARGETplus SMARTpool siRNA targeting NUSAP1 (L-004754-00-0010, Horizon, Waterbeach, Cambridge, UK), ILF2 (L-017599-00-0005, Horizon), and DHX9 (L-009950-00-0005, Horizon) were used to deplete NUSAP1, ILF2, and DHX9, respectively. ON-TARGETplus non-targeting pool (D-001810-10-20, Horizon) was used for all siControl transfections.

### 4.3. Plasmids

Full-length human NUSAP1 gene (NM_016359) was cloned into the pCMV2-Flag vector (Sigma, Burlington, MA, USA) to make an amino-terminal full-length Flag-tagged NUSAP1 (FL; Full-Length amino acids 1–441). Microtubule-binding (MT) domain (amino acids 243–367) deletion Flag-tagged NUSAP1 (ΔMT) and Charged Helical Domain (ChHD; amino acids 407–432) deletion Flag-tagged NUSAP1 (ΔChHD) plasmids were generated by Q5^®^ Site-Directed Mutagenesis Kit (New England Biolabs, Ipswich, MA, USA) according to the manufacturer’s protocol.

### 4.4. Transient Transfections and Drug Treatment

293. T and LNCaP cells were transfected with plasmids using PolyJet (SignaGen Laboratories, Frederick, MD, USA) at 3:1 (polyjet: DNA) according to the manufacturer’s instructions. Media was refreshed 24 h post-transfection. siRNAs were transfected into LNCaP cells by Lipofectamine 3000 (Invitrogen, Waltham, MA, USA), and cells were harvested 48 h post-transfection. For camptothecin (CPT) treatment experiments, cells were harvested after 48 h post-transfection of siRNA followed by additional 60 min of treatment with 10 μM CPT as previously described [41].

### 4.5. Immunoprecipitation for Mass Spectrometry Analyses

Affinity purification–mass spectrometry (AP–MS) identification of binding partners was performed as described previously [69]. Notably, 293T cells transfected with Flag-NUSAP1 were lysed in 0.2% NP-40 buffer (50 mM Tris-HCl, pH 8.0, 150 mM NaCl, 5 mM CaCl_2_, 1 mM EDTA, 0.2% NP-40, Halt protease, and phosphatase inhibitor). Lysates were homogenized 20 times using a Dounce homogenizer (pestle B) (Kimble Chase) and incubated with micrococcal nuclease (Thermo Scientific, Waltham, MA, USA). Cell extracts were collected by centrifugation at 14,000 rpm at 4 °C for 20 min. Cell extracts were incubated with antibodies (anti-Flag M2 antibody, F3165, Sigma Aldrich, Saint Louis, MO, USA) overnight at 4 °C with rotation. The next day, Protein-G Dynabeads (Invitrogen) were added to the mixture and incubated for 3 h at 4 °C with rotation. After incubation, the beads were washed three times in ice-cold wash buffer (50 mM Tris-HCl, pH 7.5, 150 mM NaCl, 1 mM EDTA, 0.05% NP-40). Proteins were eluted in acid using IgG elution buffer (Thermo Scientific) at room temperature (RT) for 10 min with gentle vortexing. The final elution was collected and neutralized with 1/10 volume of 1 M Tris-HCl, pH 9.0.

### 4.6. Mass Spectrometry (MS)

MS sample preparation and analyses were performed by the Stanford University Mass Spectrometry facility. In brief, for gel-free MS analysis, the final elutions from the immunoprecipitations were solubilized and digested using the filter-aided sample preparation (FASP) protocol [70]. Trypsin/Lys-C Mix (Promega, Madison, WI, USA) was used for protein digestion. Peptides were extracted and dried using a speed-vac prior to reconstitution and analysis. Nano reverse-phase HPLC was performed using either an Eksigent 2D nanoLC (Eksigent, Dublin, CA, USA) or Waters nanoAcquity (Waters, Milford, MA, USA) HPLC system with mobile phase A consisting of 0.1% formic acid in water and mobile phase B consisting of 0.1% formic acid in acetonitrile. A fused silica column self-packed with Duragel C18 (Peeke, Redwood City, CA, USA) matrix was used with a linear gradient from 2% B to 40% B at a flow rate of 600 nL/minute. The nanoHPLC was interfaced with a Bruker/Michrom Advance Captive spray source for nanoESI into either an LTQ Orbitrap Velos mass spectrometer (Thermo Fisher Scientific, Fremont, CA, USA) or an Orbitrap Elite (Thermo Fisher Scientific) operating in data-dependent acquisition mode to perform MS/MS on the top twelve most intense multiply charged cations.

### 4.7. Immunoprecipitation

To validate AP–MS data, cell extracts from LNCaP cells transfected with Flag-NUSAP1 were collected as described above and incubated with Ezview red Flag M2 affinity gel (Sigma Aldrich) for 4 h at 4 °C. Beads were washed three times and eluted in wash buffer containing 150 mg/mL Flag peptide (Sigma Aldrich) for 30 min at RT with a gentle vortex. For immunoblot analysis, light-chain-specific secondary antibodies (Jackson ImmunoResearch Labs, West Grove, PA, USA) were used.

### 4.8. Immunofluorescence Staining and Image Analyses

Cultured cells grown on coverslips were pre-extracted with ice-cold 0.5% Triton X-100 in PBS on ice for 5 min with gentle rocking or without pre-extraction. Cells were fixed in 4% paraformaldehyde (PFA) (Electron Microscopy Sciences) at RT for 10 min. After fixation, cells were blocked with 3% BSA in PBST at RT for 1 h. Fixed cells were incubated with the primary antibodies, NUSAP1 (NBP2-13685, Novusbio, Centennial, CO, USA), ILF2 (A303-147A, Bethyl Laboratories, Montgomery, TX, USA), DHX9 (A300-854A-M, Bethyl Laboratories), nucleolin (BNC47, Biotium, Fremont, CA, USA), and Flag (F3165, Sigma Aldrich), in the blocking buffer at 4 °C overnight, and secondary antibody, Alexa 488 (ab150113, ab150077, Abcam, Fremont, CA, USA) and Alexa 555 (a21422, Abcam), incubations at RT for 1 h. Cells were mounted with DAPI Fluoromount-G (DAPI, 4,6-diamidino-2-phenylindole) (Southern Biotech, Birmingham, AL, USA). Stained cells were imaged using Leica Application Suite X software on a Leica CTR 6500 (Leica, Wetzlar, Germany) or Zeiss LSM 880 confocal microscope. For S9.6 (gift from Karlene Cimprich, Stanford Univ.), *γ*H2AX (664039, Millipore, Burlington, MA, USA), and RNase H (M0297, NEB, Ipswich, MA, USA), immunofluorescence (IF) was carried out as described previously [41]. IF image analyses were performed as described previously [69]. Briefly, for *γ*H2AX imaging, a Leica CTR 6500 (Leica) microscope with 20X objective was used to acquire 1-3 field images (100-150 cells per sample). Nuclear fluorescence intensities were quantified with ImageJ (version 1.53p) and box-and-whisker plots were generated.

### 4.9. Protein Lysate Preparation and Immunoblotting

Protein lysates and western blotting methods were adapted from previous studies [69,71]. Briefly, cells were washed in ice-cold PBS and then were lysed in ice-cold lysis buffer [20 mM Tris-HCl, pH 7.5, 50 mM NaCl, 1% NP-40, 1% sodium deoxycholate, 2.5 mM sodium pyrophosphate, 1 mM β-glycerophosphate, 1 mM Na3VO4, 1 μg/mL leupeptin, and protease/phosphatase inhibitor cocktail (Roche)] and incubated on ice for 15 min before sonication for total protein extraction. Cell extracts were collected by centrifugation at 14,000 rpm at 4 °C for 20 min. For each sample, protein levels were measured by Pierce™ BCA Protein Assay Kit (Thermo Scientific), and equal amounts of proteins were mixed with Bolt LDS Sample Buffer (Thermo Fisher Scientific) for loading. Samples were separated by SDS-PAGE on 4–12% Bolt Bis-Tris gels (Thermo Fisher Scientific) and transferred with Bolt transfer buffer (Thermo Fisher Scientific) containing 10% methanol, 0.01% SDS using the wet transfer system (Bio-Rad, Hercules, CA, USA) onto PVDF membranes (Bio-Rad). Next, membranes were blocked with 5% non-fat milk in PBST (0.1% (*v*/*v*) Tween-20) at RT for 1 h. Samples were incubated with primary antibody at 4 °C overnight and then incubated with secondary antibody at RT for 2 h. Proteins were visualized by SuperSignal West Femto Maximum Sensitivity Substrate (Thermo Scientific), Amersham ECL Western Blotting Detection Reagent (GE), or Clarity ECL Western Blotting Substrate (Bio-Rad) and imaged using Image Lab software (Bio-Rad) on ChemiDoc XRS System (Bio-Rad). Antibodies against ILF2 (A303-147A), DHX9 (A300-854A-M), and H2AX (A300-083A-M) were from Bethyl Laboratories. Anti-NUSAP1 (NBP2-13685, Novusbio), Anti-Flag (F3165, Sigma Aldrich), and Anti-GAPDH (HRP-60004, Proteintech, Rosemont, IL, USA). Anti-HNRPC (sc-32308) and Anti-HSP 70 (sc-32239) were from Santa Cruz Biotechnology. Anti-*γ*H2AX (#9718) and anti-mouse (#7076) or rabbit (#7074) IgG HRP conjugated secondary antibodies were from Cell Signaling.

### 4.10. R-Loop Immunoprecipitation

R-loop immunoprecipitation was adapted from methods described previously [41]. LNCaP cells were washed and scraped on ice-cold TBS. Cell pellets were resuspended in hypotonic buffer [10 mM HEPES, pH 7.9, 10 mM KCl, 1.5 mM MgCl2, Halt protease and phosphatase inhibitor (Thermo Scientific)] and homogenized 10 times using a Dounce homogenizer (pestle B). The nuclei were then pelleted by centrifugation at 13,000 rpm at 4 °C for 5 min and lysed in high salt buffer [20 mM HEPES, pH 7.9, 0.42 M NaCl, 10 mM MgCl_2_, 0.5% Triton-X, Halt protease, and phosphatase inhibitor]. The nuclear extract was sonicated (Diagenode Bioruptor) for 30 s ON/OFF, 5 cycles (low amplitude), twice with 5 min incubation on ice in between. The nuclear extract was collected by centrifugation at 10,000 rpm at 4 °C for 20 min. The extract was pre-cleared with Protein-G Dynabeads for 1 h at 4 °C. After pre-clearing, the extract was diluted and supplemented to a final composition of [20 mM HEPES, pH 7.9, 50 mM NaCl, 50 mM KCl, 10 mM MgCl_2_, and 0.5% Triton-X]. For RNase H treatment, the diluted extract was incubated with 5 μL of RNase H for 3 h at 37 °C. After digestion, the extract was incubated with 5 μL of S9.6 antibody for 1.5 h at 4 °C. Meanwhile, Protein-G Dynabeads were blocked with 0.5% BSA/PBS for 1.5 h at 4 °C. After incubation, pre-blocked Dynabeads were added to the IP samples, and the samples were incubated for another 1.5 h at 4 °C. The IP samples were washed twice with wash buffer [20 mM HEPES, pH 7.9, 0.2 M NaCl, 10 mM MgCl_2_] supplemented with 0.5% Triton-X, and twice more with wash buffer alone. For elution, the beads were boiled in 2x LDS buffer with TCEP. IgG control (Jackson ImmunoResearch Labs) was generated using the same procedure.

### 4.11. R-Loop Analysis by Dot-Blot

The methods for R-loop analysis by dot-blot were adapted from those described previously [72]. Briefly, cells were washed with 1X PBS twice and lysed using cold cell lysis buffer on ice for 10 min and then centrifuged at 500× *g* for 5 min to pellet the nuclei. The supernatant was discarded and the nuclear pellet was resuspended in cold nuclear lysis buffer on ice for 10 min. Proteinase K (Qiagen, Redwood City, CA, USA) was added to the nuclear lysates, and these were incubated for 3–5 h at 55 °C, followed by the addition of elution buffer and an equal amount of phenol:chloroform:isoamyl alcohol (25:24:1 pH 8.0) (MilliporeSigma, Burlington, MA, USA) to purify genomic DNA. To demonstrate specificity of binding of ILF2, NUSAP1, and DHX9 to R-loops, RNase H (M0297, NEB) was used to digest RNA-DNA hybrids at 37 °C for 15 min. Notably, 2 µL of each sample of the untreated or RNase H treated nucleic acids was loaded at a range of concentrations onto two positively charged nylon membranes (Cytiva, North Logan, UT, USA) such that one membrane was used to assess R-loop levels with the S9.6 antibody, and the other served as a loading control by probing with the dsDNA antibody (MA1-35346, Invitrogen). The membranes were crosslinked using a UV crosslinker (Stratagene, San Diego, CA, USA) and then incubated in TBST for 1 h at RT on a shaker. The membranes were incubated with the primary antibody overnight at 4 °C and the following morning, the primary antibody was removed, and the membranes were washed 3 times with TBST. The membranes were then incubated with the secondary antibody (#7076, Cell Signaling Technology, Danvers, MA, USA) at RT for 1 h and developed with enhanced-chemiluminescence (ECL) reagents (Thermo Scientific). Membrane spot images were quantified using ImageJ (version 1.53p).

### 4.12. The Cancer Genome Atlas (TCGA) Data Analysis

NUSAP1 transcript expression levels for non-cancer and cancer samples were obtained from the prostate cancer (PRAD) TCGA dataset [48] (https://gdac.broadinstitute.org, accessed on 5 May 2022). Differences in expression between cancer and non-cancer groups were evaluated with the Mann–Whitney Test. For ILF2, NUSAP1, and DHX9, patients were grouped by RNA expression using the median as the cutoff, and disease-free survival status after surgery was visualized with Kaplan–Meier survival plots. The probability of remaining disease free was evaluated with the Log-rank test. Pearson correlation was used to evaluate the degree of correlation of gene expression between NUSAP1, ILF2, and DHX9, and the correlation between RNA expression and different Gleason scores.

### 4.13. Gene Set Enrichment Analysis (GSEA)

GSEA was performed using GSEA version 3.0 [73,74], by examining the DNA Damage Gene set (193 genes), which has been reported previously [3]. NUSAP1 was set as the phenotype label, and the ‘Metric for ranking genes’ was set for Pearson correlation. All other basic and advanced fields were set to default. Adjusted *p*-values  <  0.05 and FDR  <  0.25 were considered significant. Heatmaps were generated with the genes from GSEA software, where high vs. low NUSAP1 expression was used as the phenotype label.

### 4.14. Quantification and Statistical Analyses

Statistical analyses were performed using GraphPad Prism 9.3.1 (GraphPad Software, San Diego, CA). Unless otherwise stated, the experimental data were calculated by the following statistical analyses. For pairwise comparison, an unpaired *t*-test (parametric) or two-tailed Mann–Whitney test (nonparametric) was used. For multiple comparisons in the S9.6 signals and immunofluorescence signals, in more than two groups, a one-way ANOVA test followed by Tukey’s test was used to correct the multiple comparisons. Notably, *p*-values are indicated as *, *p* < 0.05; **, *p* < 0.01; ***, *p* < 0.001; ****, *p* < 0.0001; NS, *p* > 0.05. All experiments were performed in triplicate. Fold-enrichment and *p*-value of common interactors between NUSAP1 and ILF2, NUSAP1, and RNA/DNA hybrids interactomes were calculated from the hypergeometric distribution equation at (https://systems.crump.ucla.edu/hypergeometric/index.php, accessed on 1 August 2022) and assumed a total proteome of at least 100,000 different proteins, as previously described [75].

## Data Availability

Data: The accession number for the mass spectrometry data generated from this study is the Mass Spectrometry Interactive Virtual Environment (MassIVE) database: MSV000090440.

## Supplementary Materials

The following supporting information can be downloaded at https://www.mdpi.com/article/10.3390/ijms24076258/s1.
