# Supplementary material for: NUSAP1 Binds ILF2 to Modulate R-Loop Accumulation and DNA Damage in Prostate Cancer
Source: Int J Mol Sci. 2023 Mar 26;24(7):6258. doi: 10.3390/ijms24076258 (PMC10093842; doi:10.3390/ijms24076258)
Supplement: Supplementary file 1 [file ijms-24-06258-s001.zip › Table S1.pdf]

**Table S1** High-confidence NUSAP1 interactors from AP-MS in vehicle-treated non-synchronized 293T cells

| Gene_Name | Protein_Name | Uniprot_ID | Spectral Counts | ctrlCounts | SaintScore | BFDR |
|---|---|---|---|---|---|---|
| *NUSAP1* | Nucleolar and spindle-associated protein 1 | Q9BXS6 | 1070\|1070\|1055 | 0\|0\|0 | 1 | 0 |
| *RBMX* | RNA-binding motif protein, X chromosome | P38159 | 415\|415\|262 | 19\|25\|22 | 1 | 0 |
| *MDC1* | Mediator of DNA damage checkpoint protein 1 | Q14676 | 66\|66\|73 | 0\|0\|0 | 1 | 0 |
| *MKI67* | Proliferation marker protein Ki-67 | P46013 | 91\|91\|59 | 0\|0\|0 | 1 | 0 |
| *HP1BP3* | Heterochromatin protein 1-binding protein 3 | Q5SSJ5 | 54\|54\|55 | 0\|0\|0 | 1 | 0 |
| *RPL12* | 60S ribosomal protein L12 | P30050 | 27\|27\|23 | 1\|0\|0 | 1 | 0 |
| *WTAP* | Pre-mRNA-splicing regulator WTAP | Q15007 | 64\|64\|43 | 0\|0\|0 | 1 | 0 |
| *CHTOP* | Chromatin target of PRMT1 protein | Q9Y3Y2 | 35\|35\|42 | 0\|0\|0 | 1 | 0 |
| *STAU1* | Double-stranded RNA-binding protein Staufen homolog 1 | O95793 | 43\|43\|33 | 0\|0\|0 | 1 | 0 |
| *PSIP1* | PC4 and SFRS1-interacting protein | O75475 | 39\|39\|26 | 0\|0\|0 | 1 | 0 |
| *CCDC86* | Coiled-coil domain-containing protein 86 | Q9H6F5 | 13\|13\|16 | 0\|0\|1 | 1 | 0 |
| *IGF2BP1* | Insulin-like growth factor 2 mRNA-binding protein 1 | Q9NZI8 | 21\|21\|21 | 0\|0\|0 | 1 | 0 |
| *PINX1* | PIN2/TERF1-interacting telomerase inhibitor 1 | Q96BK5 | 29\|29\|19 | 0\|0\|0 | 1 | 0 |
| *RPS20* | 40S ribosomal protein S20 | P60866 | 25\|25\|27 | 2\|0\|1 | 1 | 0 |
| *RPS21* | 40S ribosomal protein S21 | P63220 | 25\|25\|58 | 0\|1\|2 | 1 | 0 |
| *RPL21* | 60S ribosomal protein L21 | P46778 | 34\|34\|36 | 3\|3\|4 | 1 | 0 |
| *HNRNPA0* | Heterogeneous nuclear ribonucleoprotein A0 | Q13151 | 17\|17\|19 | 0\|0\|0 | 1 | 0 |
| *ILF3* | Interleukin enhancer-binding factor 3 | Q12906 | 43\|43\|80 | 2\|0\|0 | 1 | 0 |

| | | | | | | |
|---|---|---|---|---|---|---|
| *CCDC137* | Coiled-coil domain-containing protein 137 | Q6PK04 | 17\|17\|15 | 0\|0\|0 | 1 | 0 |
| *H1-2* | Histone H1.2 | P16403 | 99\|99\|50 | 8\|6\|13 | 1 | 0 |
| *EIF6* | Eukaryotic translation initiation factor 6 | P56537 | 22\|22\|14 | 0\|0\|0 | 1 | 0 |
| *HAKAI* | E3 ubiquitin-protein ligase Hakai | Q75N03 | 13\|13\|13 | 0\|0\|0 | 1 | 0 |
| *PAIRBP1* | Plasminogen activator inhibitor 1 RNA-binding protein 1 | Q8NC51 | 14\|14\|13 | 0\|0\|0 | 1 | 0 |
| *DHX9* | DEAH box protein 9 (ATP-dependent RNA helicase A) | Q08211 | 9\|9\|45 | 0\|1\|0 | 1 | 0 |
| *DPY30* | Protein dpy-30 homolog | Q9C005 | 17\|17\|12 | 0\|0\|0 | 1 | 0 |
| *GAR1* | H/ACA ribonucleoprotein complex subunit 1 | Q9NY12 | 8\|8\|11 | 0\|0\|1 | 1 | 0 |
| *RALY* | RNA-binding protein Raly | Q9UKM9 | 9\|9\|20 | 0\|0\|0 | 1 | 0 |
| *FUS* | RNA-binding protein FUS | P35637 | 23\|23\|19 | 3\|3\|1 | 1 | 0 |
| *CIRBP* | Cold-inducible RNA-binding protein | Q14011 | 12\|12\|8 | 0\|0\|0 | 1 | 0 |
| *NOL7* | Nucleolar protein 7 | Q9UMY1 | 8\|8\|10 | 0\|0\|0 | 1 | 0 |
| *NOP10* | H/ACA ribonucleoprotein complex subunit 3 | Q9NPE3 | 9\|9\|8 | 0\|0\|0 | 1 | 0 |
| *NUPL2* | Nucleoporin-like protein 2 | O15504 | 8\|8\|15 | 0\|0\|0 | 1 | 0 |
| *RPS29* | 40S ribosomal protein S29 | P62273 | 7\|7\|7 | 0\|0\|0 | 1 | 0 |
| *THOC4* | THO complex subunit 4 | Q86V81 | 25\|25\|20 | 5\|1\|3 | 1 | 0 |
| *RPS11* | 40S ribosomal protein S11 | P62280 | 19\|19\|18 | 0\|3\|3 | 1 | 0 |
| *CBX1* | Chromobox protein homolog 1 | P83916 | 6\|6\|7 | 0\|0\|0 | 1 | 0 |
| *H1-0* | Histone H1.0 | P07305 | 6\|6\|6 | 0\|0\|0 | 1 | 0 |
| *RPL31* | 60S ribosomal protein L31 | P62899 | 22\|22\|18 | 2\|3\|5 | 1 | 0 |
| *RPL23A* | 60S ribosomal protein L23a | P62750 | 24\|24\|26 | 4\|8\|6 | 1 | 0 |
| *RPLP1* | 60S acidic ribosomal protein P1 | P05386 | 14\|14\|18 | 2\|2\|2 | 1 | 0 |
| *HNRNPCL1* | Heterogeneous nuclear ribonucleoprotein C-like 1 | O60812 | 21\|21\|16 | 2\|2\|5 | 1 | 0 |

| | | | | | | |
|---|---|---|---|---|---|---|
| *C11orf98* | Uncharacterized protein C11orf98 | E9PRG8 | 5\|5\|6 | 0\|0\|0 | 1 | 0 |
| *TIMM8B* | Mitochondrial import inner membrane translocase subunit Tim8 B | Q9Y5J9 | 7\|7\|5 | 0\|0\|0 | 1 | 0 |
| *RPL7A* | 60S ribosomal protein L7a | P62424 | 35\|35\|38 | 4\|12\|8 | 1 | 0 |
| *RPL28* | 60S ribosomal protein L28 | P46779 | 25\|25\|27 | 0\|5\|2 | 1 | 0 |
| *NHP2* | H/ACA ribonucleoprotein complex subunit 2 | Q9NX24 | 4\|4\|6 | 0\|0\|0 | 1 | 0 |
| *PHC2* | Polyhomeotic-like protein 2 | Q8IXK0 | 4\|4\|5 | 0\|0\|0 | 1 | 0 |
| *MRPS17* | 28S ribosomal protein S17 | Q9Y2R5 | 4\|4\|10 | 0\|0\|0 | 1 | 0 |
| *MRPS28* | 28S ribosomal protein S28 | Q9Y2Q9 | 4\|4\|4 | 0\|0\|0 | 1 | 0 |
| *RPL36A* | 60S ribosomal protein L36a | P83881 | 17\|17\|19 | 0\|3\|0 | 1 | 0 |
| *RPL35A* | 60S ribosomal protein L35a | P18077 | 5\|5\|4 | 0\|0\|1 | 1 | 0 |
| *CBX3* | Chromobox protein homolog 3 | Q13185 | 16\|16\|20 | 0\|4\|4 | 1 | 0 |
| *NUMA1* | Nuclear mitotic apparatus protein 1 | Q14980 | 14\|14\|3 | 0\|0\|0 | 1 | 0 |
| *RPL22* | 60S ribosomal protein L22 | P35268 | 7\|7\|3 | 0\|0\|0 | 1 | 0 |
| *RPLP0* | 60S acidic ribosomal protein P0 | P05388 | 3\|3\|13 | 0\|0\|0 | 1 | 0 |
| *RRP15* | RRP15-like protein | Q9Y3B9 | 7\|7\|3 | 0\|0\|0 | 1 | 0 |
| *RPS12* | 40S ribosomal protein S12 | P25398 | 6\|6\|3 | 0\|0\|0 | 1 | 0 |
| *SRP14* | Signal recognition particle 14 kDa protein | P37108 | 3\|3\|3 | 0\|0\|0 | 1 | 0 |
| *ZC3H8* | Zinc finger CCCH domain-containing protein 8 | Q8N5P1 | 3\|3\|3 | 0\|0\|0 | 1 | 0 |
| *RPS10L* | Putative 40S ribosomal protein S10-like | Q9NQ39 | 17\|17\|24 | 4\|4\|7 | 1 | 0 |
| *HNRNPC* | Heterogeneous nuclear ribonucleoproteins C1/C2 | P07910 | 21\|21\|33 | 6\|0\|0 | 1 | 0 |
| *H1-10* | Histone H1.10 | Q92522 | 19\|19\|17 | 0\|5\|5 | 1 | 0 |
| *RPL34* | 60S ribosomal protein L34 | P49207 | 7\|7\|7 | 0\|2\|0 | 0.99 | 0 |
| *H2AV* | Histone H2A.V | Q71UI9 | 12\|12\|11 | 0\|4\|2 | 0.99 | 0 |
| *RPS26* | 40S ribosomal protein S26 | P62854 | 3\|3\|4 | 0\|0\|1 | 0.99 | 0 |

| | | | | | | |
|---|---|---|---|---|---|---|
| *ILF2* | Interleukin enhancer-binding factor 2 | Q12905 | 2\|2\|8 | 0\|0\|0 | 0.99 | 0 |
| *KPNA2* | Importin subunit alpha-1 | P52292 | 2\|2\|9 | 0\|0\|0 | 0.99 | 0 |
| *RALYL* | RNA-binding Raly-like protein | Q86SE5 | 2\|2\|3 | 0\|0\|0 | 0.99 | 0 |
| *RPL30* | 60S ribosomal protein L30 | P62888 | 4\|4\|2 | 0\|0\|0 | 1 | 0 |
| *MRPL54* | 39S ribosomal protein L54 | Q6P161 | 2\|2\|2 | 0\|0\|0 | 0.99 | 0 |
| *RBMXL1* | RNA binding motif protein, X-linked-like-1 | Q96E39 | 2\|2\|5 | 0\|0\|0 | 0.99 | 0 |
| *RRP12* | RRP12-like protein | Q5JTH9 | 3\|3\|2 | 0\|0\|0 | 0.99 | 0 |
| *SAP30BP* | SAP30-binding protein | Q9UHR5 | 2\|2\|4 | 0\|0\|0 | 0.99 | 0 |
| *YBX3* | Y-box-binding protein 3 | P16989 | 7\|7\|2 | 0\|0\|0 | 1 | 0 |
| *ZNF346* | Zinc finger protein 346 | Q9UL40 | 4\|4\|2 | 0\|0\|0 | 1 | 0 |
| *ZNF48* | Zinc finger protein 48 | Q96MX3 | 11\|11\|2 | 0\|0\|0 | 1 | 0 |
| *RPL23* | 60S ribosomal protein L23 | P62829 | 8\|8\|9 | 0\|2\|3 | 0.99 | 0 |
| *RPS16* | 40S ribosomal protein S16 | P62249 | 11\|11\|17 | 2\|4\|4 | 0.98 | 0 |
| *RPS28* | 40S ribosomal protein S28 | P62857 | 19\|19\|6 | 3\|1\|1 | 0.98 | 0 |
| *RPL29* | 60S ribosomal protein L29 | P47914 | 29\|29\|26 | 17\|2\|1 | 0.95 | 0 |
| *HIST2H2AA* | Histone H2A type 2-A | Q6FI13 | 3\|3\|5 | 0\|1\|1 | 0.92 | 0 |
| *RPL8* | 60S ribosomal protein L8 | P62917 | 21\|21\|13 | 4\|8\|1 | 0.91 | 0 |
