# Supplementary material for: NUSAP1 Binds ILF2 to Modulate R-Loop Accumulation and DNA Damage in Prostate Cancer
Source: Int J Mol Sci. 2023 Mar 26;24(7):6258. doi: 10.3390/ijms24076258 (PMC10093842; doi:10.3390/ijms24076258)
Supplement: Supplementary file 1 [file ijms-24-06258-s001.zip › Table S2.pdf]

Table S2 High-confidence NUSAP1 interactors identified from AP-MS in nocodazole-treated 293T cells

Gene_Name	Protein_Name	Uniprot_ID	Spectral Counts	ctrlCounts	SaintScore	BFDR
NUSAP1	Nucleolar and spindle-associated protein 1	Q9BXS6	1090|1584|1197	0|0|0	1	0
RBMX	RNA-binding motif protein, X chromosome	P38159	382|359|289	19|25|22	1	0
MKI67	Proliferation marker protein Ki-67	P46013	135|117|108	0|0|0	1	0
CHTOP	Chromatin target of PRMT1 protein	Q9Y3Y2	47|147|42	0|0|0	1	0
RPL12	60S ribosomal protein L12	P30050	20|51|46	1|0|0	1	0
H1-2	Histone H1.2	P16403	73|159|95	8|6|13	1	0
HP1BP3	Heterochromatin protein 1-binding protein 3	Q5SSJ5	41|24|40	0|0|0	1	0
STAU1	Double-stranded RNA-binding protein Staufen homolog 1	O95793	20|18|27	0|0|0	1	0
RPS21	40S ribosomal protein S21	P63220	57|27|21	0|1|2	1	0
IGF2BP1	Insulin-like growth factor 2 mRNA-binding protein 1	Q9NZI8	28|13|21	0|0|0	1	0
RPL21	60S ribosomal protein L21	P46778	25|37|33	3|3|4	1	0
THOC4	THO complex subunit 4	Q86V81	29|25|25	5|1|3	1	0
RPS20	40S ribosomal protein S20	P60866	16|21|18	2|0|1	1	0
WTAP	Pre-mRNA-splicing regulator WTAP	Q15007	10|24|22	0|0|0	1	0
EIF6	Eukaryotic translation initiation factor 6	P56537	12|10|16	0|0|0	1	0
HNRNPA0	Heterogeneous nuclear ribonucleoprotein A0	Q13151	9|25|13	0|0|0	1	0
RPL28	60S ribosomal protein L28	P46779	42|53|48	0|5|2	1	0
HNRNPC	Heterogeneous nuclear ribonucleoproteins C1/C2	P07910	22|14|8	0|0|0	1	0
PAIRBP1	Plasminogen activator inhibitor 1 RNA-binding protein 1	Q8NC51	7|19|11	0|0|0	1	0
MRPS28	28S ribosomal protein S28	Q9Y2Q9	8|7|9	0|0|0	1	0

GAR1	H/ACA ribonucleoprotein complex subunit 1	Q9NY12	6|9|9	0|0|1	1	0
RPL35A	60S ribosomal protein L35a	P18077	7|9|6	0|0|1	1	0
RPS28	40S ribosomal protein S28	P62857	14|26|18	3|1|1	1	0
NOP10	H/ACA ribonucleoprotein complex subunit 3	Q9NPE3	6|7|10	0|0|0	1	0
RPS14	40S ribosomal protein S14	P62263	60|161|56	18|23|15	1	0
RPS11	40S ribosomal protein S11	P62280	23|21|16	0|3|3	1	0
CCDC137	Coiled-coil domain-containing protein 137	Q6PK04	8|13|5	0|0|0	1	0
KPNA2	Importin subunit alpha-1	P52292	5|10|10	0|0|0	1	0
PINX1	PIN2/TERF1-interacting telomerase inhibitor 1	Q96BK5	5|31|28	0|0|0	1	0
RPL22	60S ribosomal protein L22	P35268	12|7|5	0|0|0	1	0
DPY30	Protein dpy-30 homolog	Q9C005	4|7|8	0|0|0	1	0
RPLP0P6	60S acidic ribosomal protein P0-like	Q8NHW5	14|4|6	0|0|0	1	0
FUS	RNA-binding protein FUS	P35637	12|23|20	3|3|1	1	0
CBX1	Chromobox protein homolog 1	P83916	3|3|6	0|0|0	1	0
C11orf98	Uncharacterized protein C11orf98	E9PRG8	6|4|3	0|0|0	1	0
H1-0	Histone H1.0	P07305	3|9|5	0|0|0	1	0
NHP2	H/ACA ribonucleoprotein complex subunit 2	Q9NX24	3|12|6	0|0|0	1	0
NOL7	Nucleolar protein 7	Q9UMY1	7|3|4	0|0|0	1	0
MRPL54	39S ribosomal protein L54	Q6P161	3|4|4	0|0|0	1	0
RPS12	40S ribosomal protein S12	P25398	15|6|3	0|0|0	1	0
MRPS17	28S ribosomal protein S17	Q9Y2R5	13|3|5	0|0|0	1	0
TIMM8B	Mitochondrial import inner membrane translocase subunit Tim8 B	Q9Y5J9	3|7|6	0|0|0	1	0
ZNF48	Zinc finger protein 48	Q96MX3	3|8|5	0|0|0	1	0

RPL36A	60S ribosomal protein L36a	P83881	13|13|11	0|3|0	0.99	0
RPL34	60S ribosomal protein L34	P49207	7|7|8	0|2|0	0.99	0
H2AV	Histone H2A.V	Q71UI9	25|14|11	0|4|2	0.99	0
HNRNPCL1	Heterogeneous nuclear ribonucleoprotein C-like 1	O60812	11|19|22	2|2|5	0.99	0
HAKAI	E3 ubiquitin-protein ligase Hakai	Q75N03	2|16|15	0|0|0	0.99	0
NHP2L1	NHP2-like protein 1	P55769	2|6|3	0|0|0	0.99	0
RPL30	60S ribosomal protein L30	P62888	2|2|3	0|0|0	0.99	0
RPLP0	60S acidic ribosomal protein P0	P05388	7|2|7	0|0|0	0.99	0
YBX3	Y-box-binding protein 3	P16989	7|4|2	0|0|0	0.99	0
RPLP1	60S acidic ribosomal protein P1	P05386	8|10|8	2|2|2	0.98	0
RPL31	60S ribosomal protein L31	P62899	11|23|18	2|3|5	0.99	0
CBX3	Chromobox protein homolog 3	Q13185	11|10|14	0|4|4	0.96	0
CCDC86	Coiled-coil domain-containing protein 86	Q9H6F5	4|2|3	0|0|1	0.95	0
RPS26	40S ribosomal protein S26	P62854	2|2|4	0|0|1	0.92	0
RPL23	60S ribosomal protein L23	P62829	5|10|10	0|2|3	0.91	0
RPL37A	60S ribosomal protein L37a	P61513	15|22|9	0|5|4	0.9	0.01
