# Supplementary material for: NUSAP1 Binds ILF2 to Modulate R-Loop Accumulation and DNA Damage in Prostate Cancer
Source: Int J Mol Sci. 2023 Mar 26;24(7):6258. doi: 10.3390/ijms24076258 (PMC10093842; doi:10.3390/ijms24076258)
Supplement: Supplementary file 1 [file ijms-24-06258-s001.zip › Table S3.pdf]

Table S3 27 NUSAP1-interacting proteins uniquely identified in the non-synchronized cells

Gene_Name	Protein_Name
MDC1	Mediator of DNA damage checkpoint protein 1 (MDC1)
PSIP1	PC4 and SFRS1-interacting protein
ILF3	Interleukin enhancer-binding factor 3 (ILF3)
DHX9	DEAH box protein 9 (DHX9)
RALY	RNA-binding protein Raly (RALY)
CIRBP	Cold-inducible RNA-binding protein
NUPL2	Nucleoporin-like protein 2
RPS29	40S ribosomal protein S29
RPL23A	60S ribosomal protein L23a
RPL7A	60S ribosomal protein L7a
PHC2	Polyhomeotic-like protein 2
NUMA1	Nuclear mitotic apparatus protein 1
RRP15	RRP15-like protein
SRP14	Signal recognition particle 14 kDa protein
ZC3H8	Zinc finger CCCH domain-containing protein 8
RPS10L	Putative 40S ribosomal protein S10-like
H1-10	Histone H1.10
ILF2	Interleukin enhancer-binding factor 2 (ILF2)
RALYL	RNA-binding Raly-like protein
RBMXL1	RNA binding motif protein, X-linked-like-1 (RMXL1)
RRP12	RRP12-like protein
SAP30BP	SAP30-binding protein
ZNF346	Zinc finger protein 346
RPS16	40S ribosomal protein S16
RPL29	60S ribosomal protein L29
HIST2H2AA	Histone H2A type 2-A
RPL8	60S ribosomal protein L8
