# Supplementary material for: NUSAP1 Binds ILF2 to Modulate R-Loop Accumulation and DNA Damage in Prostate Cancer
Source: Int J Mol Sci. 2023 Mar 26;24(7):6258. doi: 10.3390/ijms24076258 (PMC10093842; doi:10.3390/ijms24076258)
Supplement: Supplementary file 1 [file ijms-24-06258-s001.zip › Table S4.pdf]

Table S4 NUSAP1 and ILF2 interactomes- 8 common interactors

Gene_Name	Protein_Name
DHX9	DEAH box protein 9 (DHX9)
ILF2	Interleukin enhancer-binding factor 2 (ILF2)
ILF3	Interleukin enhancer-binding factor 3 (ILF3)
HNRNPC	Heterogeneous nuclear ribonucleoproteins C1/C2 (HNRPC)
RALY	RNA-binding protein Raly (RALY)
FUS	RNA-binding protein FUS (FUS)
RBMX	RNA-binding motif protein, X chromosome (RBMX)
YBX3	Y-box-binding protein 3 (YBOX3)
