# Supplementary material for: NUSAP1 Binds ILF2 to Modulate R-Loop Accumulation and DNA Damage in Prostate Cancer
Source: Int J Mol Sci. 2023 Mar 26;24(7):6258. doi: 10.3390/ijms24076258 (PMC10093842; doi:10.3390/ijms24076258)
Supplement: Supplementary file 1 [file ijms-24-06258-s001.zip › Table S5.pdf]

Table S5 NUSAP1 and RNA:DNA Hybrids inteactomes- 33 common interactors

Gene_Name	Protein_Name
RBMX	RNA-binding motif protein, X chromosome
MKI67	Proliferation marker protein Ki-67
HP1BP3	Heterochromatin protein 1-binding protein 3
CHTOP	Chromatin target of PRMT1 protein
STAU1	Double-stranded RNA-binding protein Staufen homolog 1
PSIP1	PC4 and SFRS1-interacting protein
CCDC86	Coiled-coil domain-containing protein 86
IGF2BP1	Insulin-like growth factor 2 mRNA-binding protein 1
PINX1	PIN2/TERF1-interacting telomerase inhibitor 1
HNRNPA0	Heterogeneous nuclear ribonucleoprotein A0
ILF3	Interleukin enhancer-binding factor 3
CCDC137	Coiled-coil domain-containing protein 137
EIF6	Eukaryotic translation initiation factor 6
PAIRBP1	Plasminogen activator inhibitor 1 RNA-binding protein 1
DHX9	DEAH box protein 9 (ATP-dependent RNA helicase A) (DHX9)
GAR1	H/ACA ribonucleoprotein complex subunit 1
RALY	RNA-binding protein Raly
FUS	RNA-binding protein FUS
NOL7	Nucleolar protein 7
NOP10	H/ACA ribonucleoprotein complex subunit 3
THOC4	THO complex subunit 4
HNRNPCL1	Heterogeneous nuclear ribonucleoprotein C-like 1
NHP2	H/ACA ribonucleoprotein complex subunit 2
CBX3	Chromobox protein homolog 3
NUMA1	Nuclear mitotic apparatus protein 1
RRP15	RRP15-like protein
SRP14	Signal recognition particle 14 kDa protein
HNRNPC	Heterogeneous nuclear ribonucleoproteins C1/C2 (HNRPC)
ILF2	Interleukin enhancer-binding factor 2 (ILF2)
RBMXL1	RNA binding motif protein, X-linked-like-1
RRP12	RRP12-like protein
YBX3	Y-box-binding protein 3
HIST2H2AA	Histone H2A type 2-A
